# Arabidopsis mRNA polyadenylation machinery: comprehensive analysis of protein-protein interactions and gene expression profiling

**DOI:** 10.1186/1471-2164-9-220

**Published:** 2008-05-14

**Authors:** Arthur G Hunt, Ruqiang Xu, Balasubrahmanyam Addepalli, Suryadevara Rao, Kevin P Forbes, Lisa R Meeks, Denghui Xing, Min Mo, Hongwei Zhao, Amrita Bandyopadhyay, Lavanya Dampanaboina, Amanda Marion, Carol Von Lanken, Qingshun Quinn Li

**Affiliations:** 1 Department of Plant and Soil Sciences, University of Kentucky, Lexington, KY 40546, USA; 2 Department of Botany, Miami University, Oxford, OH 45056, USA; 3 Department of Anatomy and Neurobiology, Virginia Commonwealth University, Richmond, VA 23298, USA; 4 Sigma-Aldrich, St. Louis, MO, USA; 5 2127 Victory Palm Drive, Edgewater, FL, USA; 6 School of Public Health and Health Sciences, University of Massachusetts, Worcester, MA, USA

## Abstract

**Background:**

The polyadenylation of mRNA is one of the critical processing steps during expression of almost all eukaryotic genes. It is tightly integrated with transcription, particularly its termination, as well as other RNA processing events, i.e. capping and splicing. The poly(A) tail protects the mRNA from unregulated degradation, and it is required for nuclear export and translation initiation. In recent years, it has been demonstrated that the polyadenylation process is also involved in the regulation of gene expression. The polyadenylation process requires two components, the *cis*-elements on the mRNA and a group of protein factors that recognize the *cis*-elements and produce the poly(A) tail. Here we report a comprehensive pairwise protein-protein interaction mapping and gene expression profiling of the mRNA polyadenylation protein machinery in Arabidopsis.

**Results:**

By protein sequence homology search using human and yeast polyadenylation factors, we identified 28 proteins that may be components of Arabidopsis polyadenylation machinery. To elucidate the protein network and their functions, we first tested their protein-protein interaction profiles. Out of 320 pair-wise protein-protein interaction assays done using the yeast two-hybrid system, 56 (~17%) showed positive interactions. 15 of these interactions were further tested, and all were confirmed by co-immunoprecipitation and/or in vitro co-purification. These interactions organize into three distinct hubs involving the Arabidopsis polyadenylation factors. These hubs are centered around AtCPSF100, AtCLPS, and AtFIPS. The first two are similar to complexes seen in mammals, while the third one stands out as unique to plants. When comparing the gene expression profiles extracted from publicly available microarray datasets, some of the polyadenylation related genes showed tissue-specific expression, suggestive of potential different polyadenylation complex configurations.

**Conclusion:**

An extensive protein network was revealed for plant polyadenylation machinery, in which all predicted proteins were found to be connecting to the complex. The gene expression profiles are indicative that specialized sub-complexes may be formed to carry out targeted processing of mRNA in different developmental stages and tissue types. These results offer a roadmap for further functional characterizations of the protein factors, and for building models when testing the genetic contributions of these genes in plant growth and development.

## Background

Messenger RNA 3'-end formation is a vital step in gene expression. In this RNA processing event, a precursor mRNA is recognized, cleaved, and then polyadenylated at the free 3'-OH generated by the processing reaction (for a recent review, see [1]). This processing is directed by distinct polyadenylation signal sequences present in the substrate RNAs. These signals are recognized by an apparatus with conservation of components amongst eukaryotes. This apparatus consists of a complex of factors that control the action of poly(A) polymerases, limiting polyadenylation to RNAs containing polyadenylation signals. In mammals, these factors have been termed (by consensus) as CPSF (Cleavage and Polyadenylation Specificity Factor), CstF (Cleavage stimulatory Factor), CFI and CFII (Cleavage Factors I and II), and PAP (poly(A) polymerase)[2]. In addition, a poly(A) binding protein (PAB2) is involved in controlling the processivity of PAP as well as the final poly(A) length [3]. In yeast, 3'-end formation is mediated by a complex that also consists of several factors, each of which in turn consists of several polypeptide subunits. These include CPF (Cleavage and Polyadenylation Factor) and CF1 and 2 (Cleavage Factor 1 and 2; note the yeast CF complexes differ from the mammalian ones, and that the differences are matters of terminology and not function; [2]). While biochemical fractionation and purification has led to the designation of somewhat different complexes in various systems, for the most part, the polypeptide subunits that constitute the polyadenylation machinery in mammals and yeast (the two best-characterized systems) are strikingly conserved [4].

Messenger RNA 3'-end formation is coordinated with other steps in the course of gene expression. Several polyadenylation factor subunits interact with components of the transcription initiation machinery [5,6], and "load" onto the transcribed gene at or near the transcription initiation site [7,8]. The nuclear mRNA cap-binding complex has been reported to be involved in 3'-end processing in Hela cell extracts [9]. There is an interplay between splicing and polyadenylation that is important for determining (or defining) the 3'-terminal exon in mammalian genes [10,11]. Polyadenylation is closely linked with transcription termination [12]. Polyadenylation factor subunits also play roles in the maturation of cell-cycle-regulated histone mRNAs, snRNAs, and tRNAs [13-15]. Polyadenylation is associated with transport of mRNA from the nucleus to the cytoplasm [16,17]. Finally, associations with DNA repair and chromosome segregation have been reported [18,19]. These various observations reveal both an extensive interconnection between the polyadenylation apparatus and other processes, and a considerable potential for rearrangement and "donation" of parts of the polyadenylation complex to other processes.

The process of 3' end formation in plants is less well understood. Plant genes possess polyadenylation signals that are somewhat different from their mammalian and yeast counterparts [20-24]. In plants, three different classes of *cis*-elements are involved in mRNA 3' end formation. One of these (the "near-upstream element," or NUE) is situated between 10 and 40 nts upstream from its associated poly(A) site. The NUE is an A-rich element that may be between 6 and 10 nts in length. Another class of *cis *element (the "far-upstream element," or FUE) is located farther upstream (as far as 100 nts) from the poly(A) sites. This element resides in a similar position to efficiency elements that modulate 3' end formation in mammals and yeast [25], and bears a base composition reminiscent of downstream sequences involved in 3' end formation in mammals. The third class of *cis*-element is the poly(A) site itself and its adjacent U-rich element, the combination of these signals is now called CE or Cleavage Element [22,24].

Efforts have been made in recent years to characterize the protein factors that recognize above polyadenylation signals and forming polyadenylation complex in plants. These include the characterization of the genes and initial functional determination of the Arabidopsis homologues of PAP, CPSF and CstF subunits, and Fip1. Mutational analysis of two CPSF homologues, AtCPSF73-II and AtCPSF30, has shown that *AtCPSF73-II *is, apart from house-keeping functions, an essential gene that affects female gametophyte genetic transmission [26], and that *AtCPSF30 *is non-essential [27]. AtCPSF30 has been demonstrated to possess RNA-binding and endonuclease activity [27,28]. An Arabidopsis ortholog of FIP has been shown to bind RNA and interact in vitro with a number of other Arabidopsis polyadenylation factor subunits [29]. Two Arabidopsis CstF subunit orthologs, AtCSTF77 and AtCSTF64, interact in vitro; moreover, AtCSTF64 binds RNA [30]. Mutations in two polyadenylation-related genes (AtCPSF100 and symplekin) affect the process of posttranscriptional gene silencing [31], and mutations in another (FY) result in alterations in the timing of flowering [32].

These studies have enhanced our understanding of the plant polyadenylation factors. However, many questions remain regarding the functions of these proteins. For example, it is not clear if they exist in complexes more analogous to mammalian or yeast polyadenylation factors. Sequence-specific interactions between any of the plant proteins and polyadenylation signals have yet to be demonstrated, and interactions between the various proteins themselves have not been studied to any great extent. In addition, the integration of mRNA 3' end formation into other aspects of nuclear RNA metabolism in plants has not been studied. All of these matters are of considerable importance for the understanding of gene expression in plants.

In this paper, as an initial effort to elucidate the mechanism of mRNA polyadenylation and its role in the regulation of gene expression, we present a genome level annotation of Arabidopsis polyadenylation factors, a summary of the expression profiles of these genes, and a systematic analysis of pair-wise protein-protein interaction assays involving the Arabidopsis polyadenylation factor subunits.

## Results

### *In silico *analysis of the expression of Arabidopsis polyadenylation-related genes

The Arabidopsis genome possesses genes that encode most of the polyadenylation factor subunits that have been described in other eukaryotes (Table 1; [33]). Possible exceptions to this include the absence of orthologs to CFIm59/68 and Hrp1. However, this is probably due to an inability to identify, using BLAST, authentic orthologs in the large array of SR+RRM- or RRM-containing proteins encoded by the Arabidopsis genome. Many of these genes and their protein products have been studied previously. Moreover, with a few exceptions (discussed in the following), the expression of these genes can be seen in microarray studies. For the majority of these proteins, the sequence similarity with other eukaryotic counterparts (such as their human homologs) is extensive, suggestive of a conservation of function that has been preserved in the different eukaryotic lineages. However, a subset of the plant factors shares a more limited similarity with their eukaryotic counterparts. These proteins include AtCPSF30, AtCSTF64, and the FIPS and PCFS (Table 1). With these proteins, functional motifs are conserved, but other parts show sizable sequence divergence.

**Table 1:** Arabidopsis genes encoding plant polyadenylation factor subunits

Subunit (Mammal/yeast)	Proposed protein function in mammal or/and yeast	Arabidopsis gene (gene name)	BLASTP score against human homolog
Single genes			

CPSF160/Yhh1	Binds to AAUAAA	At5g51660 (CPSF160)	Score = 346 bits (888), Expect = 6e-93
CPSF100/Ydh1	Unknown	At5g23880 (CPSF100, ESP5)	Score = 505 bits (1301), Expect = 4e-141,
CPSF73a/Ysh1	Endonuclease	At1g61010 (CPSF73-I)	Score = 759 bits (1960), Expect = 0.0
CPSF73b/-^1^	Similar to CPSF73a	At2g01730 (CPSF73-II, FEG)	Score = 561 bits (1445), Expect = 6e-158
CPSF30/Yth1	RNA binding, endonuclease	At1g30460 (CPSF30, OXT6)	Score = 97.8 bits (242), Expect = 5e-19

CstF77/Rna14	Scaffold for CstF64 and CstF50; bridge to CPSF	At1g17760 (CSTF77)	Score = 386 bits (991), Expect = 3e-105
CstF64/Rna15	Binds to the downstream element	At1g71800 (CSTF64)	Score = 121 bits (304), Expect = 9e-26
CstF50/-^1^		At5g60940 (CSTF50)	Score = 291 bits (745), Expect = 5e-77

hPfs2/Pfs2	RNA binding	At5g13480 (FY)	Score = 394 bits (1013), Expect = 7e-108

Multiple genes			

PAP/Pap1	Creates the poly(A) tail	At1g17980 (PAPS1)	Score = 423 bits (1088), Expect = 2e-116
		At2g25850 (PAPS2)	Score = 405 bits (1040), Expect = 8e-111
		At3g06560 (PAPS3)	Score = 239 bits (611), Expect = 2e-61
		At4g32850 (PAPS4)	Score = 399 bits (1025), Expect = 4e-109

hFip1/Fip1	Interacts with PAP, regulates CPSF30 activity	At3g66652 (FIPS3)	Score = 58.2 bits (139), Expect = 3e-06
		Ag5g58040 (FIPS5)	Score = 65.1 bits (157), Expect = 3e-08*

CFIm25/-^1^	Cleavage factor; Interact with RNA	At4g25550 (CFIS2)	Score = 245 bits (625), Expect = 1e-63
		At4g29820 (CFIS1)	Score = 187 bits (474), Expect = 5e-46

hClp1/Clp1	RNA kinase	At3g04680 (CLPS3)	Score = 330 bits (845), Expect = 1e-88
		At5g39930 (CLPS5)	Score = 252 bits (643), Expect = 4e-67

hPcf11p/Pcf11	Interacts with CstF	At1g66500 (PCFS1)	Score = 57.0 bits (136), Expect = 2e-06
		At4g04885 (PCFS4)	Score = 85.9 bits (211), Expect = 1e-14
		At5g43620 (PCFS5)	Score = 57.0 bits (136), Expect = 2e-06

PabN/Pab1	Binds to poly(A), controls length of poly(A)	At5g10350 (PABN3)	Score = 122 bits (305), Expect = 2e-26,
		At5g51120 (PABN2)	Score = 119 bits (297), Expect = 2e-25
		At5g65260 (PABN1)	Score = 120 bits (302), Expect = 4e-26

Symplekin/Pta1	Part of CPSF	At5g01400 (ESP4, SYM5)	Score = 261 bits (666), Expect = 3e-67
		At1g27590 (SYM1)	Score = 54.3 bits (129), Expect = 3e-05
		At1g27595 (SYM2)	Score = 241 bits (616), Expect = 1e-61

No obvious homolog			

CFIm68/59	Unknown	none	
-/Hrp1	Part of cleavage factor in yeast	none	

* This value is from the comparison to a homologue of *Danio rerio*.

Although polyadenylation is expected to be essential for growth and development, the nature of some mutants impaired in Arabidopsis polyadenylation factor subunits [26,31,34] raises the possibility that some plant polyadenylation-related genes are active at specific times during development, or in response to particular environmental cues. To explore this hypothesis, the expression of the set of Arabidopsis genes listed in Table 1 was studied using public domain microarray data. For this study, the data available from NASC (Nottingham Arabidopsis Stock Centre) was used; normalized expression values for most of the genes listed in Table 1 was extracted from the datasets listed in Additional file 1: microarray keys and data, and plotted so as to permit easy comparison. One of the Arabidopsis polyadenylation-related genes listed in Table 1 (At4g04885, AtPCFS4) is not represented in the Affytmetrix ATH probe set and was thus not included in this study. The complete results of this study are presented in Additional file 1. The most interesting and salient aspects of this study are discussed in more detail in the following.

**Figure 1:**
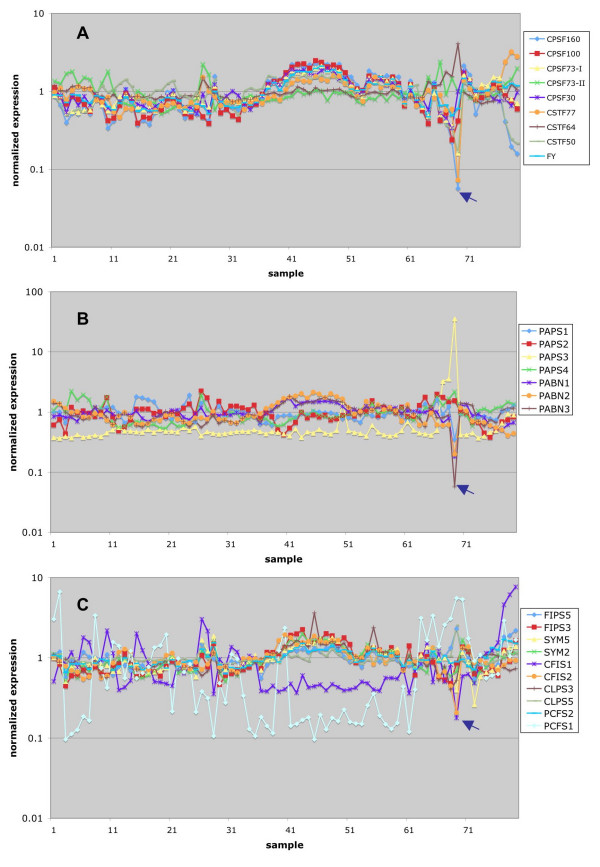
**Meta-analysis of Arabidopsis poly(A) factor gene expression during development.** Normalized expression data for the NASC Arabidopsis developmental series (Additional file 1) were extracted and plotted as shown. The set of genes listed in Table 1 were split into three groups; the grouping was done according to historical views of the polyadenylation complex. Thus, genes encoding CPSF and CSTF subunits are shown in the top panel, PAPS and PABN genes in the middle, and the remaining genes in the lower panel. This grouping also applies for the plots shown in Figures 3–5. The legends indicate the correspondence between the plots and the respective Arabidopsis gene identification designation. The numerical key for each array experiment is given along the X-axis. The full list of the keys can be found in the Additional file 1. Here is a brief description of these samples, including wt and some mutants: 1–7, root 7–21 days; 8–10, stem 7–21 days; 11–27, leaf 7–35 days; 28–38, whole plant 7–23 days; 39–49, shoot apex 7–21 days; 50–71, flowers and floral organs 21+ day; 72–79, 8 week seeds and siliques. The arrows point to the positions for mature pollen.

As shown in Figure 1, the expression of most of these genes varied modestly at different stages of growth and development. The gene encoding AtPAPS3 was a pollen-specific gene (Figure 1C). Several genes showed elevated expression in developing seeds (this pattern is typified by the AtFIPS5 and AtCSTF77 genes, respectively) while others showed reduced seed expression (AtCPSF160 is an example). Curiously, a subset of these genes showed dramatically reduced expression in pollen; this set of genes includes those encoding AtCPSF160, AtCSTF77, and AtPABN3.

**Figure 2:**
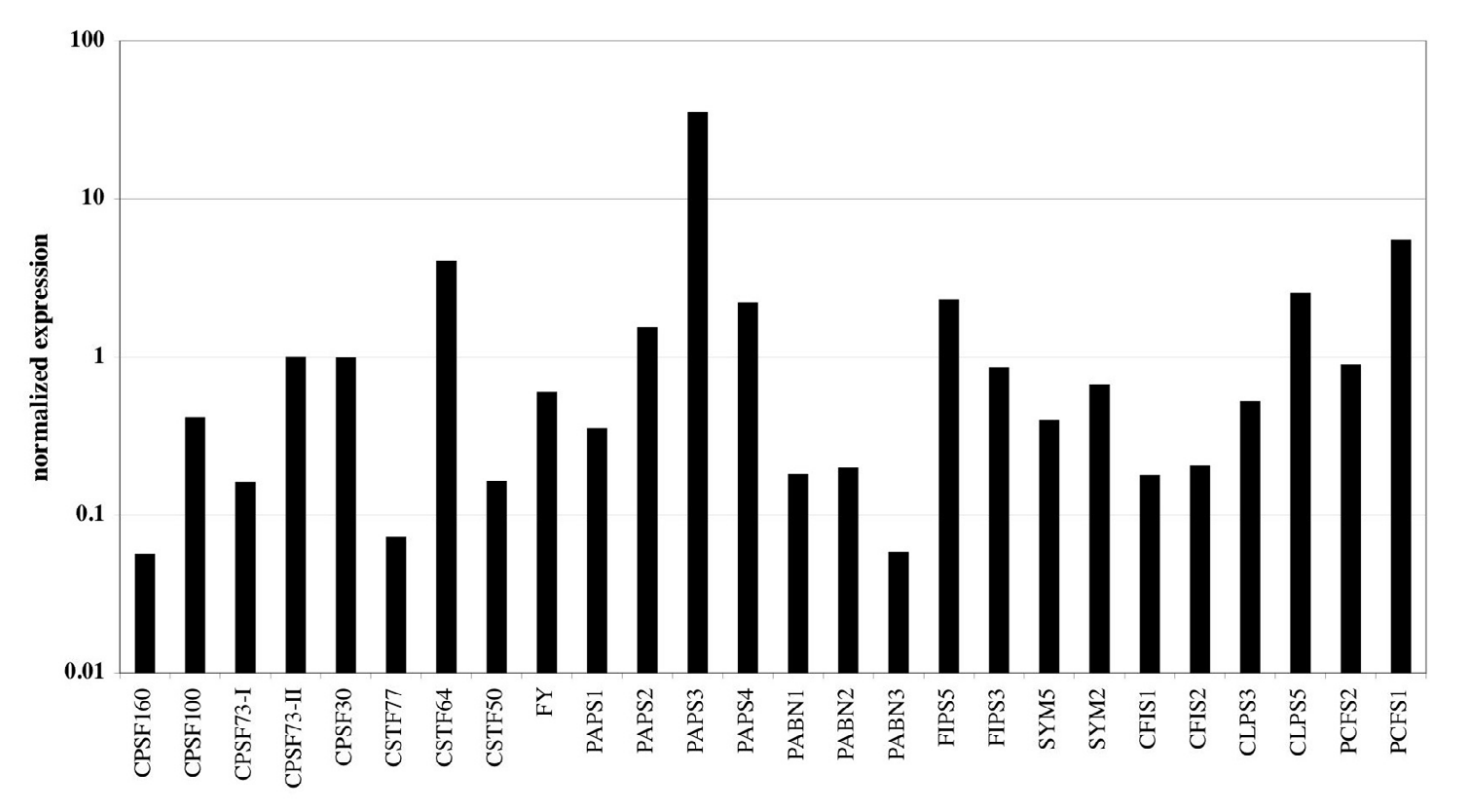
**Normalized expression of Arabidopsis polyadenylation-related genes in mature pollen.** The values for each gene in the array analysis of mature pollen were plotted as shown.

Of all the tissues and growth stages represented in Figure 1, pollen was the most different. To extend this observation, the expression of all of the genes listed in Table 1 (except for those not present in on the ATH chip) in pollen was plotted (Figure 2). This representation emphasizes the increased expression of AtPAPS3. As interesting, however, was the dramatic reduction in expression (more than 10-fold) of three other genes – AtCPSF160, AtCSTF77, and AtPABN3. Several other genes also had reduced levels of expression in pollen, suggestive of a tissue-specific gene expression program that may yield a modified polyadenylation complex.

**Figure 3:**
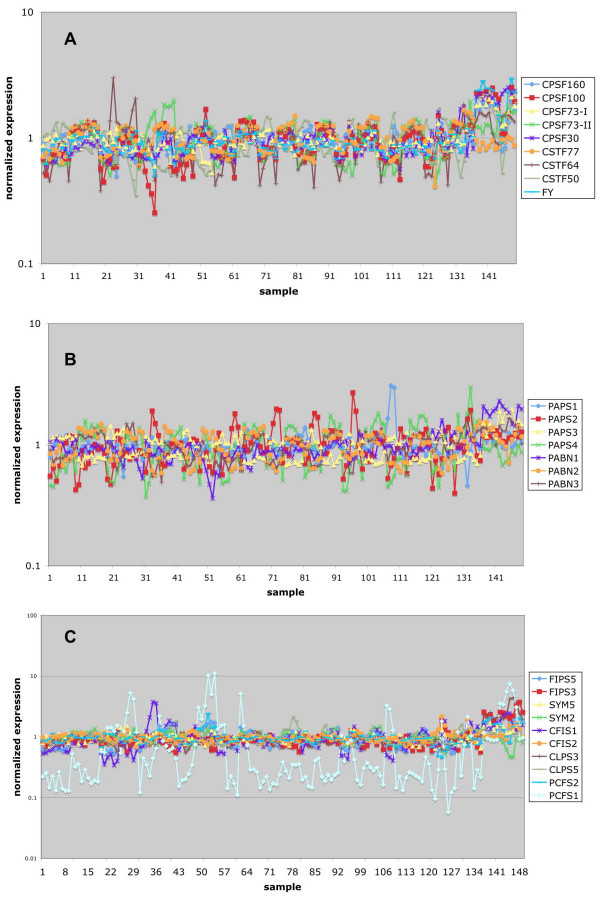
**Meta-analysis of Arabidopsis poly(A) factor gene expression in different abiotic stress conditions. **Normalized expression data for the NASC Arabidopsis abiotic stress series (Additional file 1) were extracted and plotted as shown. The legends indicate the correspondence between the plots and the respective Arabidopsis gene identification designation. The numerical key for each array experiment is given along the X-axis and the detail can be found in Additional file 1. Here is a brief list of the stress treatments: 1–18, control; 19–30, cold; 31–42, osmotic; 43–54, salt; 55–68, drought; 69–80, genotoxic; 81–92, oxidative; 93–106, UV-B; 107–120, wound; 121–136, heat; 137–141, cell culture control; 142–149, cell culture + heat.

**Figure 4:**
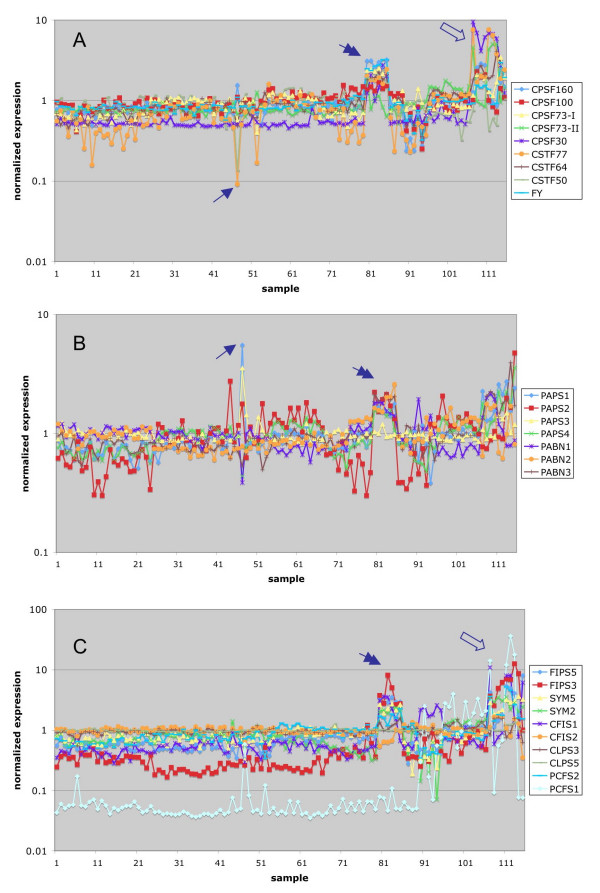
**Meta-analysis of Arabidopsis poly(A) factor gene expression in response to chemicals and hormones. **Normalized expression data for the NASC Arabidopsis chemical/hormone series (Additional file 1) were extracted and plotted as shown. The legends indicate the correspondence between the plots and the respective Arabidopsis gene identification designation. The numerical key for each array experiment is given along the X-axis, and the detail can be found in Additional file 1. The single arrows indicate the position for cycloheximide; double arrows for GA mutants; empty arrows for imbibition and ABA treatment.

A similar analysis of expression in response to various abiotic stresses revealed that most polyadenylation-related genes responded modestly, if at all, to the battery of stresses represented in the NASC dataset (Figure 3). For the most part, polyadenylation-related genes were unresponsive to chemical or hormone treatments (Figure 4). Cycloheximide, an inhibitor of protein biosynthesis, increased the expression of the AtPAPS1 and AtPAPS3 genes, suggesting that these mRNAs are relatively unstable [35]. Many of these genes were affected by mutations in giberrellic acid-related pathways and were induced by imbibition, probably reflecting induction of expression upon germination. This was most predominant with the AtFIPS3 gene, the expression of which was rather GA and imbibition-dependent.

**Figure 5:**
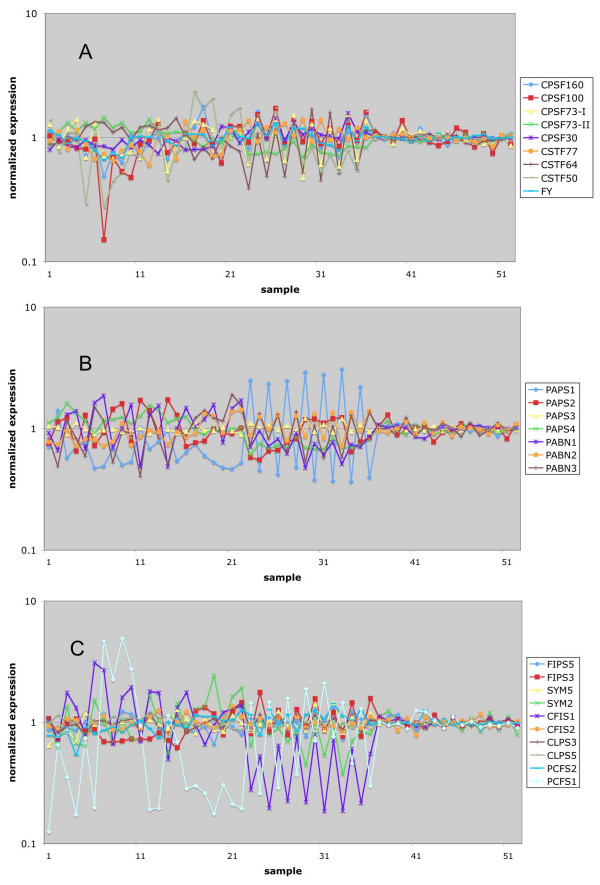
**Meta-analysis of Arabidopsis poly(A) factor gene expression in response to biotic stress and different light treatments.** Normalized expression data for the NASC Arabidopsis biotic stress series (Additional file 1) were extracted and plotted as shown. The legends indicate the correspondence between the plots and the respective Arabidopsis gene identification designation. The numerical key for each array experiment is given along the X-axis. While the full list of the agents can be found in Additional file 1, here is a brief list: 1–16, control and *Pseudomonas syringae *infection; 17–22, control and *Phytophthora *infection; 23–36, control and elicitors treatment; 37–52, dark and different light treatment.

The responses of these genes to various pathogen-related stimuli (inoculation with bacterial of fungal pathogens, treatment with elicitors of defense responses) was modest, with no poly(A) – related gene showing more than 3-fold variation in response to the different treatments (Figure 5). Dark or different light treatments had little effects on the expression of these genes (sample 37–52 in Figure 5).

### A protein-protein interaction map of the Arabidopsis polyadenylation apparatus

To better understand the functioning of the various plant polyadenylation factor subunits, a comprehensive set of pair-wise interaction assays was conducted. For this, a standard yeast two-hybrid approach was adopted. The protein coding regions for each of the Arabidopsis genes listed in Table 1 were cloned into the "AD" and "BD" yeast two-hybrid plasmids as described [34,36]. For most of these genes, the entire coding region was used. However, in some cases, the proteins were "broken" into domains, based on their predicted structures. This set of constructs (Additional file 2: Y2H constructs) was then used to collate an exhaustive pair-wise interaction map of the polyadenylation factor subunits. In these assays, both combinations of clones (e.g., AD-AtCPSF160 + BD-AtCPSF100 as well as the converse BD-AtCPSF160 + AD-AtCPSF100 combination) were tested whenever possible. Some combinations could not be tested, since several of the proteins possessed transcriptional activation domain activity in the yeast system (Additional file 2: Y2H constructs). Interactions were assessed by plating several double transformants from "non-selective" media (media that allows for identification of the double transformants) on which growth is possible only if there exists an interaction between the test subjects. All such tests included negative controls (cotransformation of the AD or BD recombinant with "empty vector" AD or BD plasmid) and positive controls [the SFN1/SFN4 combination [34], or the AtCSTF77-AtCSTF64 combination, reported as being positive [30], and confirmed in this study).

The results of this exercise are summarized in Additional file 3 (Yeast_2_Hybride_results). Of the 320 tested interactions, 56 (or 17.5%) proved to be positive. Limited confirmation tests suggest that these interactions are all authentic. Specifically, 15 independent tests, using *in vitro *or co-purification techniques, have confirmed the interactions (Table 2), and no tested two-hybrid interaction has been contradicted by other tests. Thus, the positive interactions listed in Additional file 3 are reliable.

**Table 2:** Independent confirmation of the yeast two-hybrid results

Interaction in two-hybrid assay	Confirmation method	Reference
CPSF160 – CPSF100	*In vitro *pull-down assay; Co-purification from nuclear extracts with tagged protein	[31, 34]
CPSF100 – CPSF73-I	*In vitro *pull-down assay; Co-purification from nuclear extracts with tagged protein	[31, 34]
CPSF100 – CPSF73-II	*In vitro *pull-down assay; Co-purification from nuclear extracts with tagged protein	[31, 34]
CPSF100 – CPSF30	*In vitro *pull-down assay	[34]
CPSF30 – CPSF30	In vitro copurification with recombinant proteins	[27]
CPSF100 – FY	Co-purification from nuclear extracts with tagged protein	[31]
CPSF30 – FIPS5	In vitro copurification with recombinant proteins, direct effects on enzyme activity	[28, 29]
CSTF77 – CSTF64	In vitro copurification with recombinant proteins	[30]
CSTF77 – FIPS5	In vitro copurification with recombinant proteins	[29]
CFIS1 – FIPS5	In vitro copurification with recombinant proteins	[29]
FIPS5 – PABN2	In vitro copurification with recombinant proteins	[29]
FIPS5 – PAPS4	In vitro copurification with recombinant proteins, direct effects on enzyme activity	[29]
CLPS3 – PCFS1	*In vitro *pull-down	D. Xing and QQ. Li, unpublished data (manuscript in prep)
CLPS3 –PCFS5	*In vitro *pull-down	D. Xing and QQ. Li, unpublished data (manuscript in prep)
CLPS3 – PCFS4	Affinity co-purification	[39]

**Figure 6:**
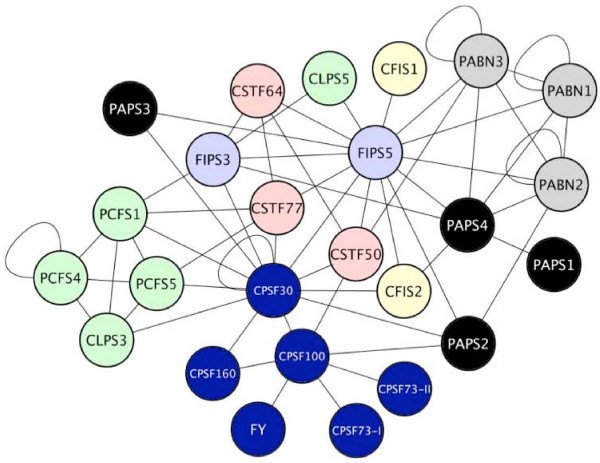
**Summary of the set of protein-protein interactions revealed by the two-hybrid assays. **Interactions were compiled and displayed using the Cytoscape software package.

The positive interactions (Additional file 3) were displayed using Cytoscape (Figure 6). From this exercise, it is apparent that the interaction network indicated by the two-hybrid study is extensively interconnected, as they are found to interact in the reciprocal yeast two-hybrid assays in most cases (e.g. AD-AtCPSF100 + BD-AtCPSF73-I, and BD-AtCPSF100 + AD-AtCPSF73-I; in some cases, due to self-activation of the BD constructs, such reciprocal tests were not possible). However, it does resolves itself into three hypothetical complexes, centered around AtFIPS5, AtCPSF100, and a putative CFIIm-like complex (consisting of AtCLPS and AtPCFS orthologs), respectively. The AtFIPS5 and AtCPSF100 subcomplexes are bridged by AtCPSF30, AtCFIS1, and three AtCSTF subunits. Additionally, AtCPSF30 links the CFIIm-like complex with the others. Interestingly, the four AtPAPS isoforms and the three AtPABN isoforms are all parts of the AtFIPS5 subcomplex, although one AtPAPS isoform (AtPAPS2) is also directly linked to the AtCPSF100 subcomplex. Also of interest, one CLPS and one CFIS isoform were positioned very differently from the other isoforms in the network. Thus, while AtCLPS3 was part of the CFIIm subcomplex, AtCLPS5 interacts independently with the two AtFIPS isoforms and with one (but only one) of the AtPAPS isoforms. While AtCFIS2 interacts with AtCPSF30, AtPAPS4, and AtFIPS5, the AtCFIS1 subunit interacts only with AtFIPS5.

**Figure 7:**
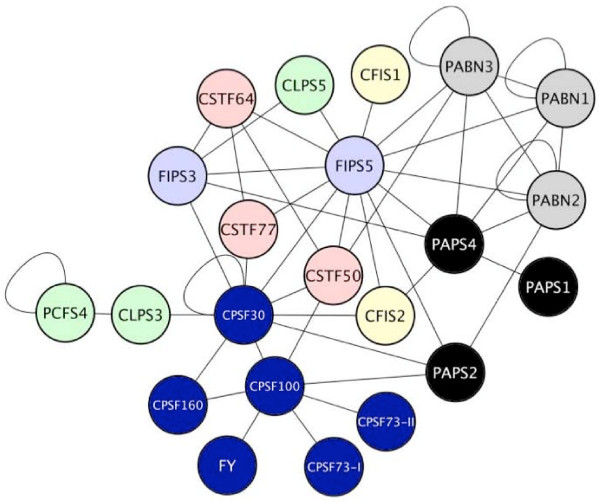
**Summary of the set of protein-protein interactions involving the products of constitutively-expressed genes.** Proteins corresponding to those genes that are expressed only in specialized tissues or times of development (PAPS3, PCFS1, and PCFS5) were removed from the network shown in Figure 6.

The results of the meta-analysis of microarray data indicate that AtPAPS3 is a pollen specific gene, that AtPCPS1 and/or AtPCPS5 are probably restricted to small parts of the plants, and that pollen and seed have a reduced polyadenylation complex. When AtPAPS3 and AtPCFS1+AtPCFS5 are removed from the overall interaction network, very little changes as far as the overall topology is concerned (Figure 7). The CFIIm-like complex reduces to but two subunits and the FIPS-PAPS hub loses one PAPS, but the general layout and inferred functionalities are otherwise unchanged. This representation is the best estimate for the network that exists in most cells in the plant.

**Figure 8:**
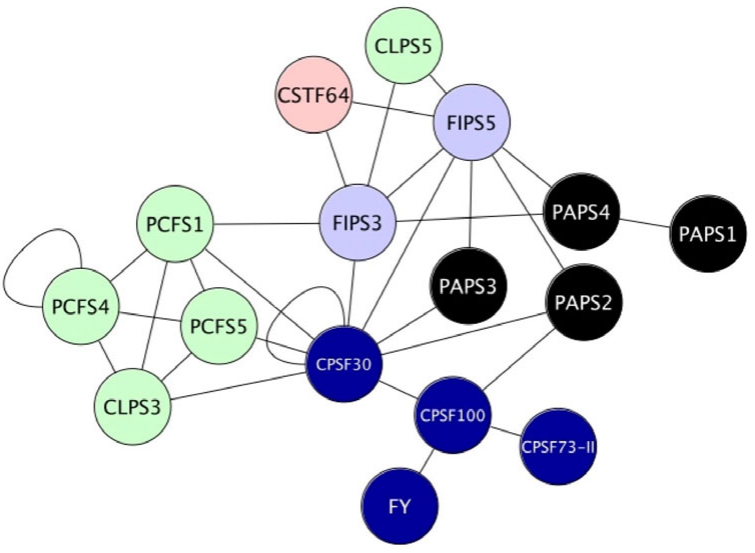
**Summary of the set of protein-protein interactions involving proteins whose genes are expressed in pollen. **Proteins whose genes are not expressed in pollen (see Figure 2) were removed from the network shown in Figure 6 and the results displayed using Cytoscape.

In contrast, the reduction of the pollen network is more substantial, as shown in Figure 8. This is apparent in the smaller size of the CPSF complex and FIPS hub. Of particular note is the absence of PABN and CFIS in the pollen network. However, these changes do not affect the overall topology of the network, which retains the CPSF and CFIIm complexes, the FIPS and CPSF30 hubs, and the bridging functions of two of the CSTF subunits.

## Discussion

### Implications of the expression characteristics of Arabidopsis genes encoding polyadenylation factor subunits

As a general rule, the expression of polyadenylation-related genes in Arabidopsis is fairly consistent over a wide range of conditions (Figures 1, 3, 4, 5). However, some interesting exceptions to this rule exist (see Figure 1). The most interesting and striking exception is the pollen-specific expression of AtPAPS3; this gene encodes a putative cytoplasmic form of PAP, and the restriction of its expression to pollen is reminiscent of the involvement of cytoplasmic PAPs in spermatogenesis in animals [37]. Interestingly, the protein encoded by AtPAPS3 is truncated with respect to the other three Arabidopsis PAPSs, as well as when compared with its eukaryotic nuclear counterparts. Moreover, this truncation leaves the protein without obvious nuclear localization signals. These observations suggest that AtPAPS3 is in fact a cytoplasmic enzyme, and plays functions during pollen development analogous to those fulfilled by the testis-specific cytoplasmic PAPs in mammals.

Two developmental states stand out when it comes to the expression of polyadenylation-related Arabidopsis genes. One of these is pollen. As noted above, one of the four Arabidopsis PAPs, AtPAPS3, is a pollen-specific gene. Remarkably, however, several other polyadenylation-related genes have normalized expression values in mature pollen that are less than 0.2 (Figures 1 and 2). These include the only genes for AtCPSF160, AtCSTF77, and the three PABN isoforms, as well as the AtCPSF73-I and AtCFIS1 genes. This observation suggests a different polyadenylation apparatus for pollen compared with other parts of the plant. Three of these subunits – AtCPSF160, AtCSTF77, and AtCPSF73-I – are core components of their respective complexes in mammals and yeast, and the prospect that polyadenylation can occur in their absence is surprising. However, removal of these seven nodes from the overall polyadenylation factor interaction network does not change the overall nature of the network in a fundamental way (Figure 8). The absence of AtCPSF160, which in mammals recognizes the AAUAAA hexamer, suggests that different polyadenylation signals are recognized in pollen compared with most other tissues in the plant. Regardless of the details, the tissue-specificity in gene expression suggests that the plant poly(A) apparatus is much more flexible than anticipated, capable of functioning with a reduced set of subunits. Of course, these considerations are predicated on the assumption that the diminished mRNA levels indicated by the microarray studies are reflected in reduced protein levels.

The other interesting developmental state is the seed. The genes encoding AtCPSF30 and AtCFIS1 have normalized expression values between 5 and 10-fold higher in seed; this is seen in several controls that study gene expression in the seed in response to ABA and imbibition (Additional file 1). This suggests a possible specialization of the polyadenylation apparatus in the seed. The possible significance of this is not clear; in other studies of the 3'-UTRs of seed-specific Arabidopsis genes, no clear nucleotide composition or sequence preference in these genes was seen (P. Thomas and A. G. Hunt, unpublished observations), apart from those that have been reported before [22]. Thus, a possible link between polyadenylation complex architecture and novel poly(A) signal usage is not indicated. The significance of the distinctive expression pattern of these two genes will have to be established by additional studies.

### Interaction network

The protein interaction network inferred from the yeast two-hybrid study resolves itself into three conceptual hubs. Two of these hubs recall biochemical studies of the polyadenylation apparatus in mammals and yeast. One hub is centered around AtCPSF100, and includes AtCPSF160, AtCPSF73-I, AtCPSF73-II, AtCPSF30, AtPAPS2, and FY. With the exception of FY (the mammalian counterpart of which has not been studied in this regard) and AtPAPS2, this hub corresponds to the classical CPSF complex, that in mammals includes CPSF160, CPSF100, CPSF73, and CPSF30. The two-hybrid results presented here are corroborated by other studies, providing a strong degree of confidence in this part of the network. Thus, the Arabidopsis CPSF subunit orthologs interact *in vitro *in a way that is consistent with the interaction network (Table 2; [34]. The four canonical plant CPSF subunits (AtCPSF160, AtCPSF100, AtCPSF73-I, and AtCPSF30) as well as AtCPSF73-II (a relative of a subunit of the recently-characterized Integrator complex; [38]) are present in nuclear extracts, indicative of their *in planta *expression and nuclear localization [31]. These proteins reside in a protein complex, as demonstrated by coimmunoprecipitation studies (R Xu and QQ Li, unpublished data; [27,31]). Interestingly, FY is part of these complexes [31], lending support to the placement of this subunit as part of CPSF in plants.

From the protein-protein interaction patterns (Figure 6), it seems that both AtCPSF73-I and AtCPSF73-II interact only with AtCPSF100 among the polyadenylation factor subunits. Moreover, they do not interact with each other or form homodimers in the two-hybrid assays, and their *in silico *expression properties show some degrees of specialized expression (Figure 1; also, [34]). These observations beg a question as to the relationship between the two AtCPSF73 orthologs. There are two possible models for their positions and functions in the complex. In one, in some tissues, both subunits are associated at the same time with AtCPSF100, in which case they are not competing for the same binding site on AtCPSF100. Alternatively, they could compete the same binding site on AtCPSF100, thus forming different complexes that exclude each other. This scenario should also apply to the tissues where these two genes are expressed differentially. Preliminary results of deletion experiments indicated that both AtCPSF73-I and II interact with the C-terminal quarter of the AtCPSF100 protein (R. Xu and QQ. Li, unpublished results), arguing for the existence of different complexes.

Symplekin was not included in the two-hybrid study, owing to some confusion at the outset of the project as to the nature of the apparent "split" gene encoding one of the two symplekin orthologs (this uncertainty remains, as discussed in [31]. However, other studies have shown that symplekin resides in a complex that includes CPSF100, CPSF160, and FY [31]; thus, symplekin would appear to be part of the CPSF complex indicated in the two-hybrid study.

Another hub that is indicated by the network analysis includes three of the PCFS isoforms and one of the CLPS orthologs. This hub corresponds to the yeast CFII complex that consists of Pcf11p and Clp1p, and to the corresponding mammalian CFII complex [2]. In other eukaryotes, Pcf11p or its homologue bridges the polyadenylation apparatus and the C-terminal domain of RNA polymerase II, thereby promoting the polyadenylation-linked termination of transcription. The CTD-interacting domain found in other eukaryotic Pcf11p proteins is present in two of the Arabidopsis orthologs [39], suggestive of a similar bridging function. Likewise, the interaction between Rna14 and Pcf11p in yeast is recapitulated with one of the Arabidopsis Pcf11p orthologs (AtPCFS5; Figure 6). However, the expression studies indicate that this interaction may only apply to the hypothetical pollen polyadenylation complex; thus, in most parts of the plant, there may be no corresponding link between the plant CFII complex and CSTF77. This is also true for the Pcf11p-Rna15 interaction that has been seen in yeast. Whether this reflects a limitation of the two-hybrid assay or divergence in the sequence and function of Pcf11 proteins is not clear. In this regard, it is possible that the CLPS-CPSF30 interaction observed in this study may serve a similar bridging function between the polyadenylation complex and the CTD of RNA polymerase II.

In mammals, hClp1 appears to be a bridge between CFIm and CPSF [40]. Such a role is not indicated by the results of this study. While the interaction of AtCLPS3 with AtCPSF30 is indicative of a link between the plant CFII complex and CPSF, there seem to be no direct physical links between either CFIS isoform and the plant CFII. Whether this discrepancy reflects limitations in the different approaches that have been used to assemble models for the polyadenylation complexes in different systems is not clear. However, resolution of the discrepancy with respect to the bridging functions of CLPS may reside in the absence of the larger CFIS subunit in the present study.

The third hub of plant polyadenylation factor subunits centers about AtFIPS5, and includes the four PAPS isoforms, the three PABN variants, and single members of the CFIS and CLPS subunit families. This hub has no obvious counterpart in the commonly-presented view of the mammalian polyadenylation apparatus (in which Fip1 is placed as part of CPSF) or in the yeast polyadenylation apparatus (in which Fip1 resides as a part of CPF). However, the interactions of the PAPS isoforms with AtFIPS5 in Arabidopsis recalls the function of Fip1 in yeast in recruiting PAP to the rest of the complex. The "FIPS hub" involves a number of proteins that are members of protein families – PAPSs and PABNs, to be specific. Moreover, with the exception of AtCSTF64, all of the interactions with AtFIPS5 involve the N-terminal 137 amino acids of the protein. It is unlikely that the sum total of interactions inferred from the two-hybrid analysis occur in a single complex; rather, a small subset of these interactions may be in force at a given moment.

Similar considerations factor into the discussion of the interactions involving AtCPSF30. While small in size, AtCPSF30 interacts with many proteins in the complex (Figure 6), including AtCPSF160, AtCPSF100, AtCFIS2, both FIPS orthologs, AtPCPS1, AtPCFS5, and AtCLPS3. It may be that AtCPSF30 is a hub around which these other subunits assemble in a large, static complex. However, AtCPSF30 seems to be too small for all these proteins to bind at once. An alternative model would involve a scenario whereby these various interactions reflect a progression through the steps of the polyadenylation reaction. These considerations reinforce those raised above, and lend themselves to a model of the plant polyadenylation complex as a dynamic system that changes its subunit composition, either as a means of recognizing different RNA substrates, interacting with other processes (such as small RNA biogenesis or transcription-related events), or progressing through the polyadenylation reaction. It is also possible that different complexes are involved alternative polyadenylation of mRNA.

Perhaps the most obvious possible difference between the predicted plant complex and the mammalian counterpart lies in the relationships of CstF subunits with other members of the complex. In mammals, CstF50 is part of an identifiable heterotrimeric complex and interacts physically with another subunit of the complex, CstF77. No such interaction is seen in the two-hybrid analysis nor in other in vitro studies [30], and the position of AtCSTF50 in the Arabidopsis network suggests that this protein is not a part of complex comparable to CSTF. AtCSTF50 does interact with CPSF, AtCSTF64, and PAPS, suggesting a novel bridging or assembly function. But such a role would seem to be different from that played by this protein in the mammalian polyadenylation complex.

The approach that we are taking can only identify the proteins that share homology with known mammalian and yeast polyadenylation factors. It is possible there are other proteins that may not share amino acid sequence homologies but functionally conserved. It is also equally possible that plants use additional proteins in the cleavage and polyadenylation process. These possibilities should be explored using different means, e.g. protein 3-D structure alignment search, proteomic and genetic approaches.

## Conclusion

Our results of mapping plant polyadenylation factor have paved the road for vigorous functional annotations of these proteins. The analysis of gene expression profiles of these genes point to formation of potential differential polyadenylation apparatus in different tissues and/or different stage of developments where specialized polyadenylation events may be warranted. The potential interacting partners combined with the gene expression profiles lay a blue print for searching differential polyadenylation machineries in various tissues and organs where alternative polyadenylation may occur.

## Methods

Arabidopsis orthologs of eukaryotic polyadenylation factor subunits were identified with BLASTP using the BLAST server of the TAIR web site [41]. For this, the Arabidopsis proteins database was queried, using the default parameters.

To conduct the *in silico *gene expression analyses (Figures 1, 2, 3, 4, 5), expression data for the Arabidopsis genes listed in Table 1 was downloaded from the NASC web site (Nottingham Arabidopsis Stock Centre;[42]). Normalized expression values were extracted, compiled (Additional file 1), and analyzed as indicated in the text and figure legends. The sample key for the experiments used here is presented in Additional file 1; this key connects the individual experiments with the various plant sample types and experimental variables.

Two hybrid assays of the interactions between the different polyadenylation factor subunits were carried out as described [29,34]. The various protein-coding regions (see Additional file 2) were subcloned into pGEM as described [27], excised as BglII fragments, and cloned into pGAD-C(1) and pGBD-C(1) [36] to yield for activation domain (AD) and binding domain (BD) clones, respectively. AD and BD plasmids were transformed into PJ69-4 [36] and dual transformants (identified as colonies growing on media lacking leucine and tryptophan, the selective markers for these two plasmids) subsequently tested on media lacking leucine, tryptophan, and adenine (the latter being one of the scorable markers for interactions). Positive interactions were those in which all tested colonies (between 4 and ten) grew on the adenine-free media. Negative controls for these tests included transformations with combinations of plasmids that included unmodified pGAD-C(1) or pGBD-C(1). For positive controls, the SFN1/SFN4 combination [34], or the AtCSTF77-AtCSTF64 combination, reported by Yao et al. [30] as being positive, were used.

Interactions were scored as either positive or negative. The set of positive interactions were compiled as .sif files and displayed using Cytoscape 2.2 [43].

## Authors' contributions

AGH and QQL were mostly responsible for the strategy and writing the manuscript. AGH did most of the microarray and Cytoscape analysis. RX, BA, SR, KPF, LM, MM, AB, LD, AM and CVL contributed to gene cloning and yeast two-hybrid assays. DX and HZ were responsible for some gene cloning, in vitro pull-down and TAP-tagged expressions. AGH, RX and DX contributed to gene homology analysis.

## Supplementary Material

Additional file 1 This file contents the keys and data that were used to produce Figure 1 to Figure 5. Click here for file

Additional file 2 This file lists all pair-wise constructs of Yeast two-hybrid assays conducted in this study. Click here for file

Additional file 3 This file contents the results of all yeast two-hybrid interaction assays conducted in this study. Click here for file
