# Supplementary material for: Arabidopsis mRNA polyadenylation machinery: comprehensive analysis of protein-protein interactions and gene expression profiling
Source: BMC Genomics. 2008 May 14;9:220. doi: 10.1186/1471-2164-9-220 (PMC2391170; doi:10.1186/1471-2164-9-220)
Supplement: Additional file 2 — This file lists all pair-wise constructs of Yeast two-hybrid assays conducted in this study. [file 1471-2164-9-220-S2.pdf]

**Hunt et al. Additional file 2 – List of two-hybrid constructs used in this study**

| Arabidopsis gene | Amino acid coordinates (complete or partial) | Two-hybrid clone(s) | reference |
|---|---|---|---|
| CPSF160 | 1-1442 (complete) | AD,BD | [1] |
| CPSF100 | 1-739 (complete) | AD,BD | [1, 2] |
| CPSF73-I | 1-693 (complete) | AD,BD | [1] |
| CPSF73-II | 1-613 (complete) | AD,BD | [1] |
| CPSF30 | 1-250 (complete) | AD | [3] |
| CSTF77 | 1-734 (complete) | AD,BD | [4] |
| CSTF77 | 406-734 (partial) | AD,BD | [4] |
| CSTF64 | 1-461 (complete) | AD,BD | [4] |
| CSTF50 | 1-429 (complete) | AD,BD | [4] |
| FY | 1-647 (complete) | AD,BD | This report |
| PAPS1 | 1-713 (complete) | AD,BD | [5] |
| PAPS2 | 1-800 (complete) | AD,BD | [2] [5] |
| PAPS3 | 26-507 (partial) | AD,BD | [5] |
| PAPS4 | 1-741 (complete) | AD,BD | [4, 5] |
| FIPS3 | 1-644 (partial*) | AD,BD | (Forbes, 2005) |
| FIPS5 | 1-137 (partial) | AD,BD | [4] |

| | | | |
|---|---|---|---|
| FIPS5 | 407-1196 (partial) | AD,BD | [4] |
| CFIS2 | 1-200 (complete) | AD,BD | This report |
| CFIS1 | 1-222 (complete) | AD,BD | This report |
| CLPS3 | 1-444 (Complete) | AD,BD | This report |
| CLPS5 | 1-423 (Complete) | AD,BD | This report |
| PCFS1 | 1-416 (Complete) | AD,BD | This report |
| PCFS4 | 1-808 (Complete) | AD,BD | This report |
| PCFS5 | 1-410 (Complete) | AD,BD | This report |
| PABN3 | 1-217 (complete) | AD,BD | This report |
| PABN2 | 1-227 (complete) | AD,BD | [4] |
| PABN1 | 1-220 (complete) | AD,BD | This report |

* - This clone carries the first 644 of the 980 amino acids that are indicated on the TAIR web site (www.arabidopsis.org). We have not been able to confirm that the portion of the clone reported here, that is confirmed as a cDNA since it spans three removed introns, is contiguous at the level of cDNA with the 3' part of the clone predicted by TAIR. We have also not been able to determine the 3' end of the cDNA reported here by 3'-RACE, despite numerous efforts.

**References:**


1. Xu RQ, Zhao HW, Dinkins RD, Cheng XW, Carberry G, Li QQ: **The 73 kD Subunit of the cleavage and polyadenylation specificity factor (CPSF) complex affects reproductive development in Arabidopsis**. *Plant Molecular Biology* 2006, **61**:799-815.
2. Elliott BJ, Dattaroy T, Meeks-Midkiff LR, Forbes KP, Hunt AG: **An interaction between an Arabidopsis poly(A) polymerase and a homologue of the 100 kDa subunit of CPSF**. *Plant Molecular Biology* 2003, **51**:373-384.
3. Delaney KJ, Xu RQ, Zhang JX, Li QQ, Yun KY, Falcone DL, Hunt AG: **Calmodulin interacts with and regulates the RNA-binding activity of an**

**Arabidopsis polyadenylation factor subunit**. *Plant Physiology* 2006, **140**:1507-1521.

4. Forbes KP, Addepalli B, Hunt AG: **An Arabidopsis Fip1 homolog interacts with RNA and provides conceptual links with a number of other polyadenylation factor subunits**. *J Biological Chemistry* 2006, **281**:176-186.
5. Meeks LR: **Isolation and Characterization of the Four Arabidopsis thaliana Poly(A) Polymerase Genes.** *Ph.D. dissertation.* Lexington: University of Kentucky 2005.
