# Supplementary material for: Arabidopsis mRNA polyadenylation machinery: comprehensive analysis of protein-protein interactions and gene expression profiling
Source: BMC Genomics. 2008 May 14;9:220. doi: 10.1186/1471-2164-9-220 (PMC2391170; doi:10.1186/1471-2164-9-220)
Supplement: Additional file 3 — This file contents the results of all yeast two-hybrid interaction assays conducted in this study. [file 1471-2164-9-220-S3.pdf]

Hunt et al. Additional file 3 Compiled results of the two-hybrid assays

Partner 1 (AtGID)	common name	Partner 2 (AtGID)	common name	results
At5g51660	CPSF160	At5g51660	CPSF160	n
At5g51660	CPSF160	At5g23880	CPSF100	y
At5g51660	CPSF160	At1g61010	CPSF73-I	n
At5g51660	CPSF160	At2g01730	CPSF73-II	n
At5g51660	CPSF160	At1g30460	CPSF30	y
At5g51660	CPSF160	At1g17760	CSTF77	n
At5g51660	CPSF160	At1g17760	CSTF77 C-terminus	n
At5g51660	CPSF160	At1g71800	CSTF64	n
At5g51660	CPSF160	At5g60940	CSTF50	n
At5g51660	CPSF160	At5g13480	FY	n
At5g51660	CPSF160	At1g17980	PAPS1	n
At5g51660	CPSF160	At2g25850	PAPS2	n
At5g51660	CPSF160	At3g06560	PAPS3	n
At5g51660	CPSF160	At4g32850	PAPS4	n
At5g51660	CPSF160	At3g66652	FIPS3	n
At5g51660	CPSF160	Ag5g58040	FIPS5 N-terminus	n
At5g51660	CPSF160	At5g58040	FIPS5 C-terminus	n
At5g51660	CPSF160	At4g29820	CFIS1	n
At5g51660	CPSF160	At4g25550	CFIS2	n
At5g51660	CPSF160	At3g04680	CLPS3	n
At5g51660	CPSF160	At5g39930	CLPS5	n
At5g51660	CPSF160	At1g66500	PCFS1	n
At5g51660	CPSF160	At4g04885	PCFS4	n
At5g51660	CPSF160	At5g43620	PCFS5	n
At5g51660	CPSF160	At5g51120	PABN1	n
At5g51660	CPSF160	At5g65260	PABN2	n
At5g51660	CPSF160	At5g10350	PABN3	n
At5g23880	CPSF100	At5g23880	CPSF100	n
At5g23880	CPSF100	At1g61010	CPSF73-I	y
At5g23880	CPSF100	At2g01730	CPSF73-II	y
At5g23880	CPSF100	At1g30460	CPSF30	y
At5g23880	CPSF100	At1g17760	CSTF77	n
At5g23880	CPSF100	At1g17760	CSTF77 C-terminus	n
At5g23880	CPSF100	At1g71800	CSTF64	n
At5g23880	CPSF100	At5g60940	CSTF50	y
At5g23880	CPSF100	At5g13480	FY	y
At5g23880	CPSF100	At1g17980	PAPS1	n
At5g23880	CPSF100	At2g25850	PAPS2	y
At5g23880	CPSF100	At3g06560	PAPS3	n
At5g23880	CPSF100	At4g32850	PAPS4	n
At5g23880	CPSF100	At3g66652	FIPS3	n
At5g23880	CPSF100	Ag5g58040	FIPS5 N-terminus	n
At5g23880	CPSF100	At5g58040	FIPS5 C-terminus	n
At5g23880	CPSF100	At4g29820	CFIS1	n
At5g23880	CPSF100	At4g25550	CFIS2	n
At5g23880	CPSF100	At3g04680	CLPS3	n
At5g23880	CPSF100	At5g39930	CLPS5	n
At5g23880	CPSF100	At1g66500	PCFS1	n

At5g23880	CPSF100	At4g04885	PCFS4	n
At5g23880	CPSF100	At5g43620	PCFS5	n
At5g23880	CPSF100	At5g51120	PABN1	n
At5g23880	CPSF100	At5g65260	PABN2	n
At5g23880	CPSF100	At5g10350	PABN3	n
At1g61010	CPSF73-I	At1g61010	CPSF73-I	n
At1g61010	CPSF73-I	At2g01730	CPSF73-II	n
At1g61010	CPSF73-I	At1g30460	CPSF30	n
At1g61010	CPSF73-I	At1g17760	CSTF77	n
At1g61010	CPSF73-I	At1g17760	CSTF77 C-terminus	n
At1g61010	CPSF73-I	At1g71800	CSTF64	n
At1g61010	CPSF73-I	At5g60940	CSTF50	n
At1g61010	CPSF73-I	At5g13480	FY	n
At1g61010	CPSF73-I	At1g17980	PAPS1	n
At1g61010	CPSF73-I	At2g25850	PAPS2	n
At1g61010	CPSF73-I	At3g06560	PAPS3	n
At1g61010	CPSF73-I	At4g32850	PAPS4	n
At1g61010	CPSF73-I	At3g66652	FIPS3	n
At1g61010	CPSF73-I	Ag5g58040	FIPS5 N-terminus	n
At1g61010	CPSF73-I	At5g58040	FIPS5 C-terminus	n
At1g61010	CPSF73-I	At4g29820	CFIS1	n
At1g61010	CPSF73-I	At4g25550	CFIS2	n
At1g61010	CPSF73-I	At3g04680	CLPS3	n
At1g61010	CPSF73-I	At5g39930	CLPS5	n
At1g61010	CPSF73-I	At1g66500	PCFS1	n
At1g61010	CPSF73-I	At4g04885	PCFS4	n
At1g61010	CPSF73-I	At5g43620	PCFS5	n
At1g61010	CPSF73-I	At5g51120	PABN1	n
At1g61010	CPSF73-I	At5g65260	PABN2	n
At1g61010	CPSF73-I	At5g10350	PABN3	n
At2g01730	CPSF73-II	At2g01730	CPSF73-II	n
At2g01730	CPSF73-II	At1g30460	CPSF30	n
At2g01730	CPSF73-II	At1g17760	CSTF77	n
At2g01730	CPSF73-II	At1g17760	CSTF77 C-terminus	n
At2g01730	CPSF73-II	At1g71800	CSTF64	n
At2g01730	CPSF73-II	At5g60940	CSTF50	n
At2g01730	CPSF73-II	At5g13480	FY	n
At2g01730	CPSF73-II	At1g17980	PAPS1	n
At2g01730	CPSF73-II	At2g25850	PAPS2	n
At2g01730	CPSF73-II	At3g06560	PAPS3	n
At2g01730	CPSF73-II	At4g32850	PAPS4	n
At2g01730	CPSF73-II	At3g66652	FIPS3	n
At2g01730	CPSF73-II	Ag5g58040	FIPS5 N-terminus	n
At2g01730	CPSF73-II	At5g58040	FIPS5 C-terminus	n
At2g01730	CPSF73-II	At4g29820	CFIS1	n
At2g01730	CPSF73-II	At4g25550	CFIS2	n
At2g01730	CPSF73-II	At3g04680	CLPS3	n
At2g01730	CPSF73-II	At5g39930	CLPS5	n
At2g01730	CPSF73-II	At1g66500	PCFS1	n
At2g01730	CPSF73-II	At4g04885	PCFS4	n
At2g01730	CPSF73-II	At5g43620	PCFS5	n
At2g01730	CPSF73-II	At5g51120	PABN1	n

At2g01730	CPSF73-II	At5g65260	PABN2	n
At2g01730	CPSF73-II	At5g10350	PABN3	n
At1g30460	CPSF30	At1g30460	CPSF30	y
At1g30460	CPSF30	At1g17760	CSTF77	y
At1g30460	CPSF30	At1g17760	CSTF77 C-terminus	y
At1g30460	CPSF30	At1g71800	CSTF64	n
At1g30460	CPSF30	At5g60940	CSTF50	y
At1g30460	CPSF30	At5g13480	FY	n
At1g30460	CPSF30	At1g17980	PAPS1	n
At1g30460	CPSF30	At2g25850	PAPS2	y
At1g30460	CPSF30	At3g06560	PAPS3	y
At1g30460	CPSF30	At4g32850	PAPS4	a
At1g30460	CPSF30	At3g66652	FIPS3	y
At1g30460	CPSF30	Ag5g58040	FIPS5 N-terminus	y
At1g30460	CPSF30	At5g58040	FIPS5 C-terminus	n
At1g30460	CPSF30	At4g29820	CFIS1	n
At1g30460	CPSF30	At4g25550	CFIS2	y
At1g30460	CPSF30	At3g04680	CLPS3	y
At1g30460	CPSF30	At5g39930	CLPS5	n
At1g30460	CPSF30	At1g66500	PCFS1	y
At1g30460	CPSF30	At4g04885	PCFS4	y
At1g30460	CPSF30	At5g43620	PCFS5	n
At1g30460	CPSF30	At5g51120	PABN1	n
At1g30460	CPSF30	At5g65260	PABN2	n
At1g30460	CPSF30	At5g10350	PABN3	n
At1g17760	CSTF77	At1g17760	CSTF77	n
At1g17760	CSTF77	At1g17760	CSTF77 C-terminus	nt
At1g17760	CSTF77	At1g71800	CSTF64	y
At1g17760	CSTF77	At5g60940	CSTF50	n
At1g17760	CSTF77	At5g13480	FY	n
At1g17760	CSTF77	At1g17980	PAPS1	n
At1g17760	CSTF77	At2g25850	PAPS2	n
At1g17760	CSTF77	At3g06560	PAPS3	n
At1g17760	CSTF77	At4g32850	PAPS4	n
At1g17760	CSTF77	At3g66652	FIPS3	n
At1g17760	CSTF77	Ag5g58040	FIPS5 N-terminus	y
At1g17760	CSTF77	At5g58040	FIPS5 C-terminus	n
At1g17760	CSTF77	At4g29820	CFIS1	n
At1g17760	CSTF77	At4g25550	CFIS2	n
At1g17760	CSTF77	At3g04680	CLPS3	n
At1g17760	CSTF77	At5g39930	CLPS5	n
At1g17760	CSTF77	At1g66500	PCFS1	y
At1g17760	CSTF77	At4g04885	PCFS4	n
At1g17760	CSTF77	At5g43620	PCFS5	y
At1g17760	CSTF77	At5g51120	PABN1	n
At1g17760	CSTF77	At5g65260	PABN2	n
At1g17760	CSTF77	At5g10350	PABN3	n
At1g17760	CSTF77 C-terminus	At1g17760	CSTF77 C-terminus	nt
At1g17760	CSTF77 C-terminus	At1g71800	CSTF64	y
At1g17760	CSTF77 C-terminus	At5g60940	CSTF50	n
At1g17760	CSTF77 C-terminus	At5g13480	FY	n
At1g17760	CSTF77 C-terminus	At1g17980	PAPS1	n

At1g17760	CSTF77 C-terminus	At2g25850	PAPS2	n
At1g17760	CSTF77 C-terminus	At3g06560	PAPS3	n
At1g17760	CSTF77 C-terminus	At4g32850	PAPS4	n
At1g17760	CSTF77 C-terminus	At3g66652	FIPS3	n
At1g17760	CSTF77 C-terminus	Ag5g58040	FIPS5 N-terminus	y
At1g17760	CSTF77 C-terminus	At5g58040	FIPS5 C-terminus	n
At1g17760	CSTF77 C-terminus	At4g29820	CFIS1	n
At1g17760	CSTF77 C-terminus	At4g25550	CFIS2	n
At1g17760	CSTF77 C-terminus	At3g04680	CLPS3	n
At1g17760	CSTF77 C-terminus	At5g39930	CLPS5	n
At1g17760	CSTF77 C-terminus	At1g66500	PCFS1	n
At1g17760	CSTF77 C-terminus	At4g04885	PCFS4	n
At1g17760	CSTF77 C-terminus	At5g43620	PCFS5	y
At1g17760	CSTF77 C-terminus	At5g51120	PABN1	n
At1g17760	CSTF77 C-terminus	At5g65260	PABN2	n
At1g17760	CSTF77 C-terminus	At5g10350	PABN3	n
At1g71800	CSTF64	At1g71800	CSTF64	n
At1g71800	CSTF64	At5g60940	CSTF50	y
At1g71800	CSTF64	At5g13480	FY	n
At1g71800	CSTF64	At1g17980	PAPS1	n
At1g71800	CSTF64	At2g25850	PAPS2	n
At1g71800	CSTF64	At3g06560	PAPS3	n
At1g71800	CSTF64	At4g32850	PAPS4	n
At1g71800	CSTF64	At3g66652	FIPS3	y
At1g71800	CSTF64	Ag5g58040	FIPS5 N-terminus	n
At1g71800	CSTF64	At5g58040	FIPS5 C-terminus	y
At1g71800	CSTF64	At4g29820	CFIS1	n
At1g71800	CSTF64	At4g25550	CFIS2	n
At1g71800	CSTF64	At3g04680	CLPS3	n
At1g71800	CSTF64	At5g39930	CLPS5	n
At1g71800	CSTF64	At1g66500	PCFS1	n
At1g71800	CSTF64	At4g04885	PCFS4	n
At1g71800	CSTF64	At5g43620	PCFS5	n
At1g71800	CSTF64	At5g51120	PABN1	n
At1g71800	CSTF64	At5g65260	PABN2	n
At1g71800	CSTF64	At5g10350	PABN3	n
At5g60940	CSTF50	At5g60940	CSTF50	n
At5g60940	CSTF50	At5g13480	FY	n
At5g60940	CSTF50	At1g17980	PAPS1	n
At5g60940	CSTF50	At2g25850	PAPS2	n
At5g60940	CSTF50	At3g06560	PAPS3	n
At5g60940	CSTF50	At4g32850	PAPS4	n
At5g60940	CSTF50	At3g66652	FIPS3	n
At5g60940	CSTF50	Ag5g58040	FIPS5 N-terminus	y
At5g60940	CSTF50	At5g58040	FIPS5 C-terminus	n
At5g60940	CSTF50	At4g29820	CFIS1	n
At5g60940	CSTF50	At4g25550	CFIS2	n
At5g60940	CSTF50	At3g04680	CLPS3	n
At5g60940	CSTF50	At5g39930	CLPS5	n
At5g60940	CSTF50	At1g66500	PCFS1	n
At5g60940	CSTF50	At4g04885	PCFS4	n
At5g60940	CSTF50	At5g43620	PCFS5	n

At5g60940	CSTF50	At5g51120	PABN1	n
At5g60940	CSTF50	At5g65260	PABN2	n
At5g60940	CSTF50	At5g10350	PABN3	y
At5g13480	FY	At5g13480	FY	n
At5g13480	FY	At1g17980	PAPS1	n
At5g13480	FY	At2g25850	PAPS2	n
At5g13480	FY	At3g06560	PAPS3	n
At5g13480	FY	At4g32850	PAPS4	n
At5g13480	FY	At3g66652	FIPS3	n
At5g13480	FY	Ag5g58040	FIPS5 N-terminus	n
At5g13480	FY	At5g58040	FIPS5 C-terminus	n
At5g13480	FY	At4g29820	CFIS1	n
At5g13480	FY	At4g25550	CFIS2	n
At5g13480	FY	At3g04680	CLPS3	n
At5g13480	FY	At5g39930	CLPS5	n
At5g13480	FY	At1g66500	PCFS1	n
At5g13480	FY	At4g04885	PCFS4	n
At5g13480	FY	At5g43620	PCFS5	n
At5g13480	FY	At5g51120	PABN1	n
At5g13480	FY	At5g65260	PABN2	n
At5g13480	FY	At5g10350	PABN3	n
At1g17980	PAPS1	At1g17980	PAPS1	n
At1g17980	PAPS1	At2g25850	PAPS2	n
At1g17980	PAPS1	At3g06560	PAPS3	n
At1g17980	PAPS1	At4g32850	PAPS4	y
At1g17980	PAPS1	At3g66652	FIPS3	n
At1g17980	PAPS1	Ag5g58040	FIPS5 N-terminus	n
At1g17980	PAPS1	At5g58040	FIPS5 C-terminus	n
At1g17980	PAPS1	At4g29820	CFIS1	n
At1g17980	PAPS1	At4g25550	CFIS2	n
At1g17980	PAPS1	At3g04680	CLPS3	n
At1g17980	PAPS1	At5g39930	CLPS5	n
At1g17980	PAPS1	At1g66500	PCFS1	n
At1g17980	PAPS1	At4g04885	PCFS4	n
At1g17980	PAPS1	At5g43620	PCFS5	n
At1g17980	PAPS1	At5g51120	PABN1	n
At1g17980	PAPS1	At5g65260	PABN2	n
At1g17980	PAPS1	At5g10350	PABN3	n
At2g25850	PAPS2	At2g25850	PAPS2	n
At2g25850	PAPS2	At3g06560	PAPS3	n
At2g25850	PAPS2	At4g32850	PAPS4	n
At2g25850	PAPS2	At3g66652	FIPS3	n
At2g25850	PAPS2	Ag5g58040	FIPS5 N-terminus	y
At2g25850	PAPS2	At5g58040	FIPS5 C-terminus	n
At2g25850	PAPS2	At4g29820	CFIS1	n
At2g25850	PAPS2	At4g25550	CFIS2	n
At2g25850	PAPS2	At3g04680	CLPS3	n
At2g25850	PAPS2	At5g39930	CLPS5	n
At2g25850	PAPS2	At1g66500	PCFS1	n
At2g25850	PAPS2	At4g04885	PCFS4	n
At2g25850	PAPS2	At5g43620	PCFS5	n
At2g25850	PAPS2	At5g51120	PABN1	n

At2g25850	PAPS2	At5g65260	PABN2	y
At2g25850	PAPS2	At5g10350	PABN3	n
At3g06560	PAPS3	At3g06560	PAPS3	n
At3g06560	PAPS3	At4g32850	PAPS4	n
At3g06560	PAPS3	At3g66652	FIPS3	n
At3g06560	PAPS3	Ag5g58040	FIPS5 N-terminus	y
At3g06560	PAPS3	At5g58040	FIPS5 C-terminus	n
At3g06560	PAPS3	At4g29820	CFIS1	n
At3g06560	PAPS3	At4g25550	CFIS2	n
At3g06560	PAPS3	At3g04680	CLPS3	n
At3g06560	PAPS3	At5g39930	CLPS5	n
At3g06560	PAPS3	At1g66500	PCFS1	n
At3g06560	PAPS3	At4g04885	PCFS4	n
At3g06560	PAPS3	At5g43620	PCFS5	n
At3g06560	PAPS3	At5g51120	PABN1	n
At3g06560	PAPS3	At5g65260	PABN2	n
At3g06560	PAPS3	At5g10350	PABN3	n
At4g32850	PAPS4	At4g32850	PAPS4	n
At4g32850	PAPS4	At3g66652	FIPS3	y
At4g32850	PAPS4	Ag5g58040	FIPS5 N-terminus	y
At4g32850	PAPS4	At5g58040	FIPS5 C-terminus	n
At4g32850	PAPS4	At4g29820	CFIS1	n
At4g32850	PAPS4	At4g25550	CFIS2	y
At4g32850	PAPS4	At3g04680	CLPS3	n
At4g32850	PAPS4	At5g39930	CLPS5	n
At4g32850	PAPS4	At1g66500	PCFS1	n
At4g32850	PAPS4	At4g04885	PCFS4	n
At4g32850	PAPS4	At5g43620	PCFS5	n
At4g32850	PAPS4	At5g51120	PABN1	y
At4g32850	PAPS4	At5g65260	PABN2	y
At4g32850	PAPS4	At5g10350	PABN3	y
At3g66652	FIPS3	At3g66652	FIPS3	n
At3g66652	FIPS3	Ag5g58040	FIPS5 N-terminus	y
At3g66652	FIPS3	At5g58040	FIPS5 C-terminus	n
At3g66652	FIPS3	At4g29820	CFIS1	n
At3g66652	FIPS3	At4g25550	CFIS2	n
At3g66652	FIPS3	At3g04680	CLPS3	n
At3g66652	FIPS3	At5g39930	CLPS5	y
At3g66652	FIPS3	At1g66500	PCFS1	y
At3g66652	FIPS3	At4g04885	PCFS4	n
At3g66652	FIPS3	At5g43620	PCFS5	n
At3g66652	FIPS3	At5g51120	PABN1	n
At3g66652	FIPS3	At5g65260	PABN2	n
At3g66652	FIPS3	At5g10350	PABN3	n
Ag5g58040	FIPS5 N-terminus	Ag5g58040	FIPS5 N-terminus	n
Ag5g58040	FIPS5 N-terminus	At5g58040	FIPS5 C-terminus	n
Ag5g58040	FIPS5 N-terminus	At4g29820	CFIS1	y
Ag5g58040	FIPS5 N-terminus	At4g25550	CFIS2	y
Ag5g58040	FIPS5 N-terminus	At3g04680	CLPS3	n
Ag5g58040	FIPS5 N-terminus	At5g39930	CLPS5	n
Ag5g58040	FIPS5 N-terminus	At1g66500	PCFS1	n
Ag5g58040	FIPS5 N-terminus	At4g04885	PCFS4	n

Ag5g58040	FIPS5 N-terminus	At5g43620	PCFS5	n
Ag5g58040	FIPS5 N-terminus	At5g51120	PABN1	y
Ag5g58040	FIPS5 N-terminus	At5g65260	PABN2	y
Ag5g58040	FIPS5 N-terminus	At5g10350	PABN3	y
At5g58040	FIPS5 C-terminus	At5g58040	FIPS5 C-terminus	n
At5g58040	FIPS5 C-terminus	At4g29820	CFIS1	n
At5g58040	FIPS5 C-terminus	At4g25550	CFIS2	n
At5g58040	FIPS5 C-terminus	At3g04680	CLPS3	n
At5g58040	FIPS5 C-terminus	At5g39930	CLPS5	n
At5g58040	FIPS5 C-terminus	At1g66500	PCFS1	n
At5g58040	FIPS5 C-terminus	At4g04885	PCFS4	n
At5g58040	FIPS5 C-terminus	At5g43620	PCFS5	n
At5g58040	FIPS5 C-terminus	At5g51120	PABN1	n
At5g58040	FIPS5 C-terminus	At5g65260	PABN2	n
At5g58040	FIPS5 C-terminus	At5g10350	PABN3	n
At4g29820	CFIS1	At4g29820	CFIS1	n
At4g29820	CFIS1	At4g25550	CFIS2	n
At4g29820	CFIS1	At3g04680	CLPS3	n
At4g29820	CFIS1	At5g39930	CLPS5	n
At4g29820	CFIS1	At1g66500	PCFS1	n
At4g29820	CFIS1	At4g04885	PCFS4	n
At4g29820	CFIS1	At5g43620	PCFS5	n
At4g29820	CFIS1	At5g51120	PABN1	n
At4g29820	CFIS1	At5g65260	PABN2	n
At4g29820	CFIS1	At5g10350	PABN3	n
At4g25550	CFIS2	At4g25550	CFIS2	n
At4g25550	CFIS2	At3g04680	CLPS3	n
At4g25550	CFIS2	At5g39930	CLPS5	n
At4g25550	CFIS2	At1g66500	PCFS1	n
At4g25550	CFIS2	At4g04885	PCFS4	n
At4g25550	CFIS2	At5g43620	PCFS5	n
At4g25550	CFIS2	At5g51120	PABN1	n
At4g25550	CFIS2	At5g65260	PABN2	n
At4g25550	CFIS2	At5g10350	PABN3	n
At3g04680	CLPS3	At3g04680	CLPS3	n
At3g04680	CLPS3	At5g39930	CLPS5	n
At3g04680	CLPS3	At1g66500	PCFS1	y
At3g04680	CLPS3	At4g04885	PCFS4	y
At3g04680	CLPS3	At5g43620	PCFS5	y
At3g04680	CLPS3	At5g51120	PABN1	n
At3g04680	CLPS3	At5g65260	PABN2	n
At3g04680	CLPS3	At5g10350	PABN3	n
At5g39930	CLPS5	At5g39930	CLPS5	n
At5g39930	CLPS5	At1g66500	PCFS1	n
At5g39930	CLPS5	At4g04885	PCFS4	n
At5g39930	CLPS5	At5g43620	PCFS5	n
At5g39930	CLPS5	At5g51120	PABN1	n
At5g39930	CLPS5	At5g65260	PABN2	n
At5g39930	CLPS5	At5g10350	PABN3	n
At1g66500	PCFS1	At1g66500	PCFS1	n
At1g66500	PCFS1	At4g04885	PCFS4	y
At1g66500	PCFS1	At5g43620	PCFS5	y

At1g66500	PCFS1	At5g51120	PABN1	n
At1g66500	PCFS1	At5g65260	PABN2	n
At1g66500	PCFS1	At5g10350	PABN3	n
At4g04885	PCFS4	At4g04885	PCFS4	y
At4g04885	PCFS4	At5g43620	PCFS5	y
At4g04885	PCFS4	At5g51120	PABN1	n
At4g04885	PCFS4	At5g65260	PABN2	n
At4g04885	PCFS4	At5g10350	PABN3	n
At5g43620	PCFS5	At5g43620	PCFS5	n
At5g43620	PCFS5	At5g51120	PABN1	n
At5g43620	PCFS5	At5g65260	PABN2	n
At5g43620	PCFS5	At5g10350	PABN3	n
At5g51120	PABN1	At5g51120	PABN1	y
At5g51120	PABN1	At5g65260	PABN2	y
At5g51120	PABN1	At5g10350	PABN3	y
At5g65260	PABN2	At5g65260	PABN2	y
At5g65260	PABN2	At5g10350	PABN3	y
At5g10350	PABN3	At5g10350	PABN3	y
